# The *ACTN3* R577X Polymorphism Is Associated with Cardiometabolic Fitness in Healthy Young Adults

**DOI:** 10.1371/journal.pone.0130644

**Published:** 2015-06-24

**Authors:** Chelsea L. Deschamps, Kimberly E. Connors, Matthias S. Klein, Virginia L. Johnsen, Jane Shearer, Hans J. Vogel, Joseph M. Devaney, Heather Gordish-Dressman, Gina M. Many, Whitney Barfield, Eric P. Hoffman, William E. Kraus, Dustin S. Hittel

**Affiliations:** 1 Department of Biochemistry and Molecular Biology, Cumming School of Medicine, University of Calgary, 2500 University Drive, Calgary, Alberta, T2N 1N4, Canada; 2 Faculty of Kinesiology, University of Calgary, 2500 University Drive, Calgary, Alberta, T2N 1N4, Canada; 3 Children’s National Medical Center, 111 Michigan Ave NW, Washington DC, United States of America; 4 Duke University, 304 Research Drive, Durham, NC, United States of America; Victoria University, AUSTRALIA

## Abstract

Homozygosity for a premature stop codon (X) in the *ACTN3* “sprinter” gene is common in humans despite the fact that it reduces muscle size, strength and power. Because of the close relationship between skeletal muscle function and cardiometabolic health we examined the influence of *ACTN3 R577X* polymorphism over cardiovascular and metabolic characteristics of young adults (n = 98 males, n = 102 females; 23 ± 4.2 years) from our Assessing Inherent Markers for Metabolic syndrome in the Young (AIMMY) study. Both males and females with the RR vs XX genotype achieved higher mean VO_2_ peak scores (47.8 ± 1.5 vs 43.2 ±1.8 ml/O_2_/min, p = 0.002) and exhibited higher resting systolic (115 ± 2 vs 105 ± mmHg, p = 0.027) and diastolic (69 ± 3 vs 59 ± 3 mmHg, p = 0.005) blood pressure suggesting a role for *ACTN3* in the maintenance of vascular tone. We subsequently identified the expression of alpha-actinin 3 protein in pulmonary artery smooth muscle, which may explain the genotype-specific differences in cardiovascular adaptation to acute exercise. In addition, we utilized targeted serum metabolomics to distinguish between RR and XX genotypes, suggesting an additional role for the *ACTN3* R577X polymorphism in human metabolism. Taken together, these results identify significant cardiometabolic effects associated with possessing one or more functional copies of the *ACTN3* gene.

## Introduction

The sarcomeric α-actinins play an important role in generating skeletal muscle contractions by stabilizing actin thin filaments within the myofibrillar array [1, 2]. In human skeletal muscle α-actinin-2 (encoded by the *ACTN2 gene*) is expressed in all muscle fiber types whereas α-actinin-3 (*ACTN3*) is expressed in a subset of fast-twitch glycolytic muscle fibers where it contributes to the generation of rapid contractions [1]. Uniquely, α-actinin-3 expression is completely absent in 18–20% of the global population because of a common nonsense polymorphism in the *ACTN3* gene wherein arginine (R) is converted into a stop codon (X) at residue 577 [3]. While the frequency of the *ACTN3* 577X null allele varies significantly between populations, at least one copy of the *ACTN3* R577 allele is beneficial for participation in sprint and power sports [4] whereas the *ACTN3* 577XX genotype is overrepresented in some endurance athlete cohorts [5] [6]. While numerous studies have confirmed associations between the *ACTN3* R577 allele and muscle power phenotypes in humans (i.e sprint time, fiber type) there have been far fewer studies linking the *ACTN3* 577X allele with endurance phenotypes [5]. Notwithstanding these observations *ACTN3* knockout mice exhibit increased recovery from exercise fatigue and a shift towards slow twitch or endurance type skeletal muscle metabolism [7]. It is hypothesized that a shift towards a more efficient metabolism may be responsible for driving the (relatively) recent positive selection of the *ACTN3* 577X allele, which arose 40,000–60,000 years ago when anatomically modern humans migrated out of Africa [8–11].

Recently a number of studies have described a role for *ACTN3* R577X in the aging process and all-cause mortality in humans [12, 13]. Notably, the R577 allele has been associated with increased survival times in patients with chronic heart failure [14] as well as increased bone mineral density/decreased fracture risk [15] suggesting an extra-sarcomeric role for α-actinin-3. While much of the research on *ACTN3* has focused on skeletal muscle phenotypes related to power/strength driven sports [16] there have been relatively few studies identifying associations with specific components of cardiovascular and metabolic fitness [12, 17]. Herein we describe the identification of novel associations between the *ACTN3* R577X polymorphism, cardiovascular fitness and metabolite biomarkers in healthy young adults.

## Materials and Methods

### Participants

This study was approved by the Conjoint Health Research Ethics Board at the University of Calgary (Ethics ID: E23521) and is registered under the clinicaltrials.gov identifier NCT00966407. Written informed consent was obtained from all subjects before participation in any testing. All subjects (n = 98 males, n = 102 females) were a part of the Assessing Inherited Markers of Metabolic Syndrome in the Young (AIMMY) Study described previously [18]. Briefly subjects were: (1) between the ages of 18 and 35 years; (2) had completed puberty; and (3) willing and able to provide informed consent. Recruitment occurred at the University of Calgary (UCalgary) main campus using posters, information on campus wide monitors, brief classroom sessions and the university’s website. All eligible, consenting participants were considered to be healthy at the time of enrolment. Health was defined as an absence of: (1) evidence of clinically relevant systemic disease associated with disorders of glucose metabolism; (2) chronic use of glucocorticoid or appetite suppressants; (3) the use of drugs that alter glucose metabolism or other medications known to alter blood levels being tested in this study (ie statins); (4) previous diagnosis or treatment for any hematologic-oncologic disorder; (5) history or current treatment for an eating disorder; (6) current treatment for weight loss; (7) history of bariatric surgery; (8) history of neurosurgical procedure.

### Blood Measures

Blood samples were collected in de-identified tubes after an 8–12 hour, overnight fast. Blood for lipoprotein assays (LDL-C, High-Density Lipoprotein Cholesterol (HDL-C), Total Cholesterol (TC), and Triglycerides (TG)) as well as insulin, glucose, C-reactive protein (CRP), and HbA1c was collected using serum stopper tubes containing a clot activator and a silicon gel separator. After collection, samples were spun at 3000 rpm for 10 minute and stored at 2–8°C at UCalgary until being transported to Calgary Lab Services (Calgary, AB) for analysis as described previously [18, 19].

### Genotyping

Genomic DNA for genetic analysis was isolated from peripheral blood as described previously [18]. Blood samples were collected in tubes containing an ethylene diamine tetra-ascetic acid (EDTA) anticoagulant and were stored at 2–8°C for a maximum of one week before being sent to the Children’s National Medical Centre (CNMC) in Washington, DC without subject identification. The ACTN3 R577X SNP (rs1815739) was identified using TaqMan allele discrimination assay [20].

### Cardiovascular Fitness Assessment

Family history, ethnicity, diet and physical activity levels were recorded by self-report using secure online questionnaires and an iPad as described previously [18, 19]. Hypertension was defined as BP ≥ 140/90 mmHg at two separate time points. Body mass index (BMI) was calculated by dividing the subjects height in meters by their weight in kg^2^. Percent body fat (%BF) and bone mineral density (BMD) was measured using a dual-energy x-ray absorptiometry scan (DXA) (Hologic QDR 4500A scanner, Hologic Inc, Walthan, MA.). Resting heart rate, resting systolic (SBP) and diastolic (DBP) blood pressure, and grip strength (via an Almedic 100kg hand grip dynamometer in the UC cohort (Almedic, Montreal, QC, Canada) were performed. VO_2_peak was assessed using the Bruce treadmill protocol as an indicator of cardiovascular fitness [21].

### Immunoblotting

Pre-made smooth muscle cell blots (cat. # TB53) were purchased from GBiosciences (St. Louis, MO). Briefly, proteins (50 ug) isolated from smooth muscle cells were solubilized in SDS-lysis buffer and electrophoresed in a 10 well, 4–20% SDS-polyacrylamide gradient gel, followed by electroblotting onto PVDF membrane. Blots were washed with 0.05% Tween-PBS (PBST) and blocked with 5% milk in PBST for 1 hour at room temperature. For examining α-actinin 3 expression a well-characterized polyclonal antibody (a gift from A. Beggs) directed against a region within amino acids 1 and 363 of ACTN3, was used at a 1:1000 dilution and incubated overnight at 4 degrees Celsius. Membranes were washed in PBST buffer and incubated with Goat anti-rabbit secondary antibody (Cell Signaling, Beverly, MA) conjugated to horseradish peroxidase. All blots were subsequently stripped and re-probed with an beta-actin antibody (Cell Signaling) as a loading control as described previously [22].To show the specificity of the α-actinin 3 primary antibody, previously genotyped human skeletal muscle samples (50 ug each) from the STRRIDE study [23] were similarly probed with each antibody. Signals were detected using enhanced chemiluminescence substrate (ThermoScientific, Rockford, IL) and chemiluminescence was digitally captured and quantified using the Chemigenius^2^ BioImaging System (Syngene, Frederick, MD).

### Statistical Analysis

All statistical analyses were performed using SPSS Statistics, version 20 (IBM). All data are presented as mean ± SEM. All subjects included an additive genetic model (RR vs. RX vs. XX) used age, sex and ethnicity (Caucasian vs. all others) tested as possible covariates. All models were adjusted for age and sex (unless sex stratified). Analysis of covariance with the Sidak method for post-hoc multiple comparisons adjustment was used for normally distributed outcomes. Quantile regression with the Sidak method for post-hoc multiple comparisons adjustment was used for non-normally distributed outcomes (% Body Fat and CRP) as described previously [24]. All resulting adjusted means are shown as transformed values as those are the numbers used for statistical models. No adjustment for multiple testing was done on the p-values reported here. We tested a minimum of 18 outcomes (considering the total cohort, females only, and males only as separate analyses). This leaves us with a multiple testing adjusted nominal significance level of 0.003; i.e. only p-value ≤0.003 would be considered statistically significant is taking into account the numbers of statistical tests performed. In addition, Pearson correlation coefficients were calculated and the resulting p-values were corrected for multiple testing using false discovery rate (FDR) controlling at a 20% level [25].

### LC-MS Metabolite Measurements and Data Analysis

As described in previous studies [26], serum samples were analyzed at Chenomx (Edmonton, AB) using LC-MS *AbsoluteIDQ p150* kits (Biocrates Life Sciences, Innsbruck, Austria) employing stable-isotope labeled internal standards. Acylcarnitines, amino acids, sugars, phosphatidylcholines (PCs) and sphingomyelins (SMs) were quantified. As individual sugars were not separated by this method, the hexose values were determined as the sum of all individual sugars. Before statistical analyses, values below the lower limit of quantification (LLOQ) or above the upper limit of quantification (ULOQ) were discarded. For metabolites with only semi quantitative values available, the LLOQ was conservatively estimated as 10 times the limit of detection (LOD), as previously suggested.[Guidelines for the validation and verification of quantitative and qualitative test methods; National Association of Testing Authorities, Australia: Silverwater, NSW, Australia, 2013.] Metabolites with greater than 20% missing values (due to being below LLOQ or above ULOQ) were excluded from analysis. This rendered 87 out of originally 148 metabolites for analysis, including 30 diacyl PCs, 27 acyl-alkyl PCs, 7 lysoPCs, 8SMs, 5 hydroxySMs, 8 amino acids acetylcarnitine and hexose. Missing values were imputed with the half minimum observed value of the respective metabolite. Metabolomics statistical analyses were performed in R 3.02 (R Foundation for Statistical Computing, Vienna, Austria). Support Vector Machines with linear kernels were used to classify RR (CC) and XX (TT) samples as described previously [26]. SVM have been previously shown to be robust classifiers with superior classification performance for metabolomics data sets [27]. To create a balanced sample set for optimal SVM training, a subset of RR samples was randomly chosen to match the number of XX samples. SVM training was repeated for 10 different sets of randomly chosen RR samples, and the average accuracy was calculated. Classification performance was assessed using leave-one-out cross validation. For feature selection, metabolites were sorted according to Mann-Whitney U-test p-values, and SVMs were trained for different numbers of metabolites, and the number with maximum accuracy was chosen.

## Results

### Subject Characteristics

Two hundred participants (mean age = 23 ± 4.2 y; 98 males and 102 females) from the University of Calgary student population completed all three visits as part of the AIMMY study [18]. The majority (80%) were Caucasian; remaining participants were Asian (16.7%) and Egyptian (3.3%). As a means of ensuring genotyping accuracy we analyzed our AIMMY cohort and found the ACTN3 R577X locus to be in Hardy-Weinberg Equilibrium (HWE) (Table 1) with (p(R) = 0.558; p(X) = 0.442; P = 0.54) as described previously [28].

**Table 1:** ACTN3 R577X Allele Frequencies and Hardy-Weinberg Equilibrium.

Genotype	Observed frequency	Expected frequency	p(R)	p(X)	p-value
RR	60	62	0.558	0.442	0.54
RX	103	99			
XX	37	39			

### Fitness Associations

We identified significant associations between *ACTN3* genotype, systolic (SBP, p = 0.027) and diastolic blood pressure (DBP, p = 0.005) and VO_2_ peak (P = 0.002) where all individuals with the RR genotype achieved greater mean values than those with the RX or XX genotypes (Tables 2, 3). When considering gender differences, the effects of *ACTN3* genotype on VO_2_peak were driven primarily by our female subjects whereas the effect in males approached statistical significance p = 0.052 (RR vs RX). Similarly, the association of *ACTN3* genotype (RR vs RX) with peak HR was only significant in males and not in females or the combined AIMMY cohort. The association of *ACTN3* genotype with SBP (RR vs XX) was only significant in the combined cohort. This phenomenon, combining two non-significant cohorts to produce a significant effect is known as Simpson’s paradox, and is the result of cohort traits (i.e. gender differences) that exhibit very different means [29] [26]. There were no significant differences in grip strength max score between *ACTN3* genotypes.

**Table 2:** Association between *ACTN3* genotype and cardiometabolic traits.

Outcome	Total cohort with age and sex adjustment	Females only with age adjustment	Males only with age adjustment
**Anthropometric**			
Height (cm)	0.22	0.93	0.08
Weight (kg)	0.38	0.0390 *	0.17
BMI	0.57	0.0153 *	0.17
BMD	0.99	0.48	0.99
% Body Fat	0.06	0.0125 *	0.16
**Cardiometabolic**			
Glucose (mg/dL)	0.27	0.19	0.16
Triglycerides (mg/dL)	0.79	0.75	0.22
Total cholesterol (mg/dL)	0.60	0.86	0.24
HDL (mg/dL)	0.09	0.19	0.22
LDL (mg/dL)	0.13	0.54	0.12
Insulin (uIU/mL)	0.74	0.29	0.69
Hba1c	0.24	0.64	0.31
CRP	0.50	0.62	0.62
**Fitness & Strength**			
Max score (strength)	0.40	0.90	0.46
VO_2_Peak (ml/kg/min)	0.002 *	0.0128 *	0.0523
Peak HR (bpm)	0.45	0.07	0.0139 *
SBP (mmHg)	0.027*	0.14	0.43
DBP (mmHg)	0.005 *	0.0316 *	0.0002 *

Shown are *ACTN3* genotype effect p-values for each anthropometric, cardiometabolic, fitness and strength variable measured in the University of Calgary AIMMY cohort.

* p-values < 0.05

**Table 3:** Significant associations between all *ACTN3* genotypes.

Outcome	Cohort	Covariate	Genotype p-value	N; adjusted mean ± SEM
VO_2_Peak (ml/kg/min)	All	Age, sex	0.002	RR (N = 58; 47.8 ± 1.5) *, ** RX (N = 97; 43.9 ± 1.4) * XX (N = 33; 43.2 ± 1.8) **
DBP (mmHg)	All	Age, sex	0.005	RR (N = 58; 69 ± 3)* RX (N = 97; 69 ± 3)** XX (N = 33; 59 ± 3)*, **
SBP (mmHg)	All	Age, sex	0.027	RR (N = 58; 115 ± 2) * RX (N = 97; 112 ± 2) XX (N = 33; 105 ± 2) *
Peak HR (bpm)	Males	Age	0.0139	RR (N = 30; 189 ± 4) * RX (N = 46; 195 ± 4) * XX (N = 17; 189 ± 4)
% Body Fat (DXA)	Females	Age	0.0125	RR (N = 29; 24.11 ± 6.48) * RX (N = 54; 23.55 ± 4.83) ** XX (N = 19; 29.07 ± 6.46) *, **
BMI	Females	Age	0.0153	RR (N = 29; 22.9 ± 0.7) RX (N = 54; 22.1 ± 0.7) * XX (N = 19; 24.5 ± 0.9) *
Weight (kg)	Females	Age	0.0390	RR (N = 29; 61.8 ± 2.4) RX (N = 54; 59.8 ± 2.1) * XX (N = 19; 66.7 ± 2.9) *

Shown are adjusted mean values ± SEM for significant traits where * and ** denote significant differences (p<0.05) between mean genotype values identified by post-hoc analysis.

### Anthropometric Associations

In female subjects only, total body weight and BMI were consistently and significantly higher (p<0.05) in RX vs XX individuals (Tables 2 & 3). A broader association was observed with DXA derived %BF values between RR and XX, as well as RX vs XX women respectively. As with VO_2_peak the effect of *ACTN3* genotype on body composition parameters were driven primarily by females.

### ACTN3 Expression in Smooth Muscle Cells

**Fig 1:**
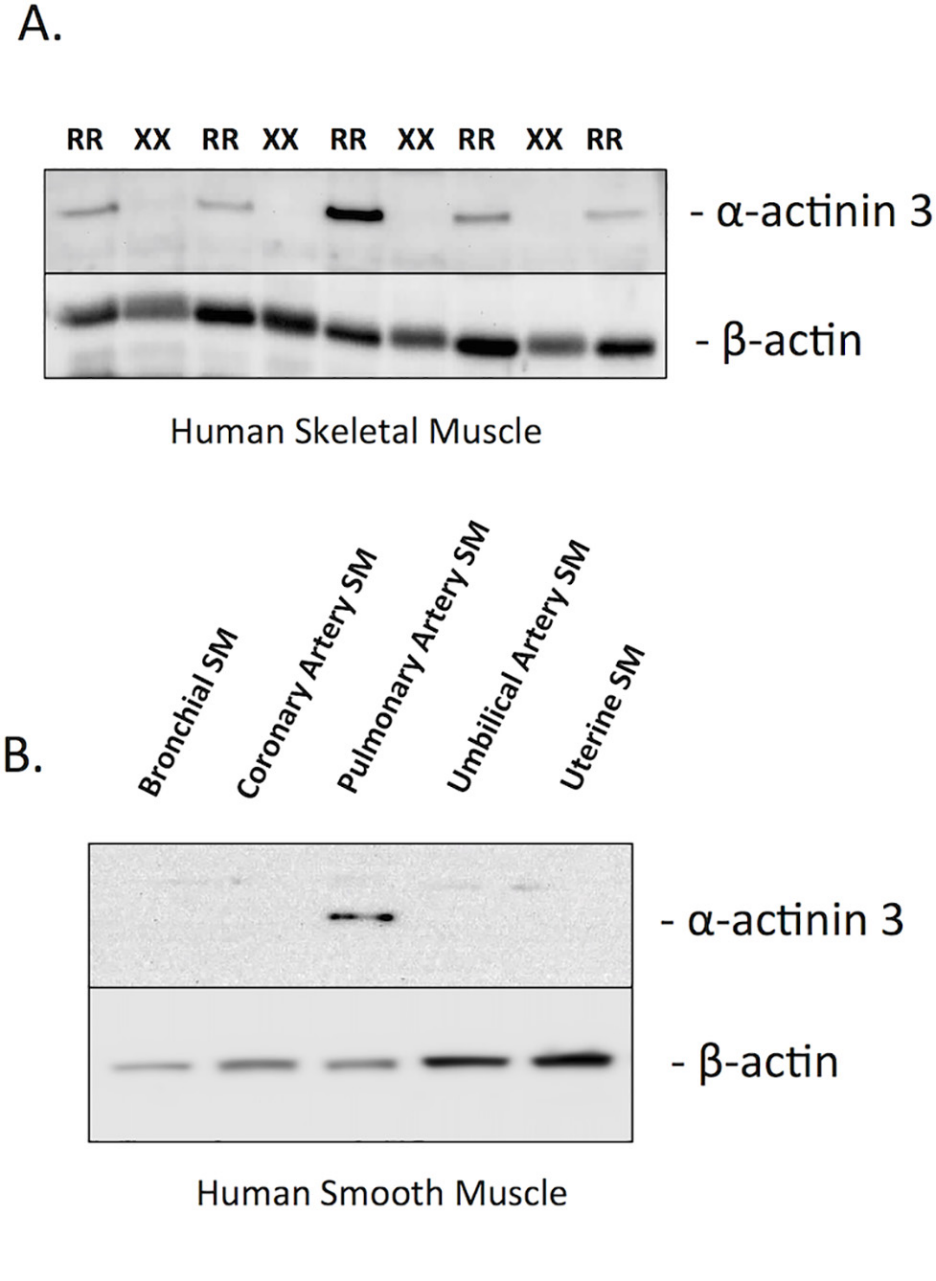
The expression of alpha-actinin-3 in human skeletal muscle and pulmonary artery smooth muscle. Shown are **A)** A rabbit polyclonal alpha-actinin 3 antibody probed against skeletal muscle samples from *ACTN3* RR577 and 577XX individuals from the STRRIDE Study [30]. **B**) This same antibody probed against human smooth muscle (SM) cell panel. Each lane contains 50 ug of protein extract, which were stripped and reprobed with a beta-actin antibody as a loading control.

**Fig 2:**
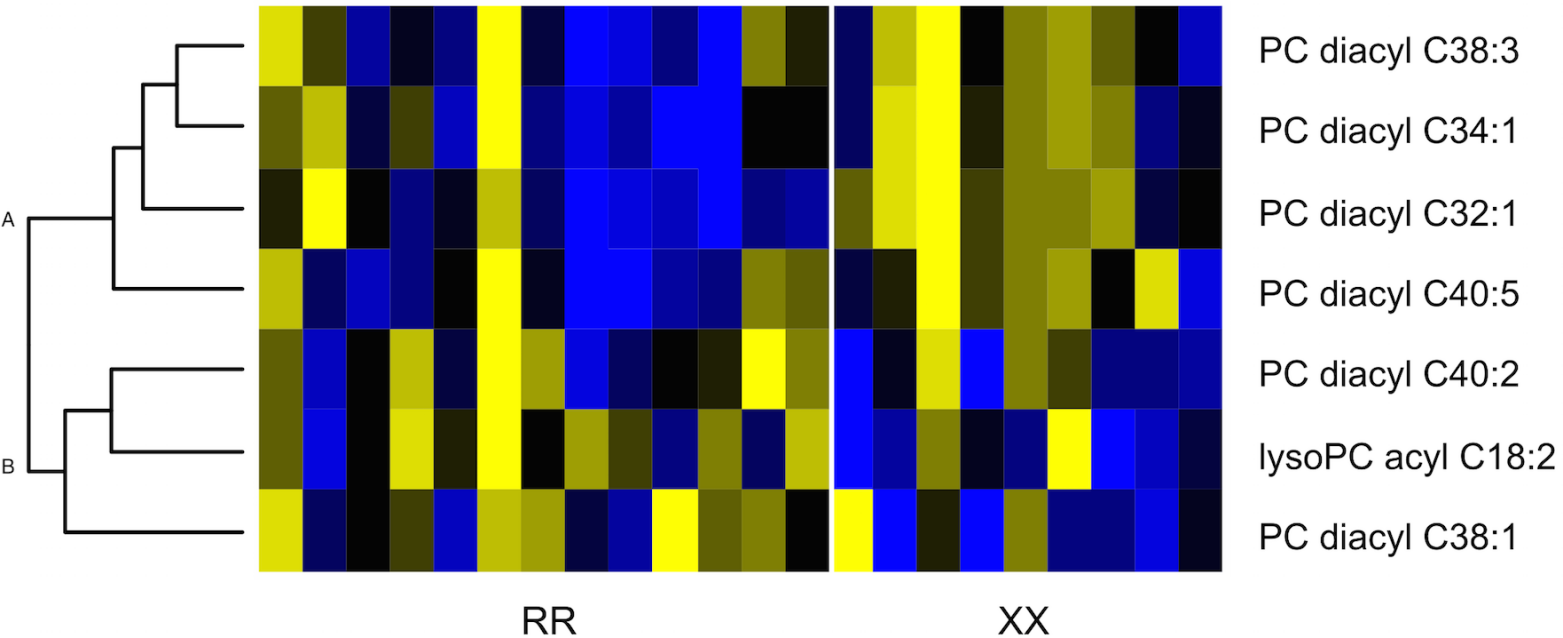
Heatmap and hierarchical clustering of significantly different metabolites stratified by *ACTN3* RR vs XX genotype. Yellow blocks represent high concentrations; blue blocks represent low concentrations; black blocks represent medium concentrations.

The α-actinin 3 polyclonal antibody was first validated against previously genotyped skeletal muscle samples from the STRRIDE study (Fig 1A) which served as both positive (RR) and negative (XX) controls [30]. A ~100 kDa band corresponding to the calculated molecular weight of mature α-actinin 3 was observed in RR but not XX skeletal muscle (Fig 2A). A ~100 kDa band was also observed in pulmonary artery smooth muscle cells but not in smooth muscle cells from Bronchiole, Coronary Artery, Umbilical Artery or Uterus (Fig 1B). The normalized (to B-actin) expression level of α-actinin 3 in pulmonary artery smooth muscle was ~ 5 x lower than in skeletal muscle.

### Cardio-metabolomic Analysis

We have recently found success in identifying in novel genotype-metabolite associations using metabolomic analysis coupled to support vector machine (SVM) analysis [26]. SVMs have been consistently shown to outperform commonly used classification approaches such as partial least-squares discriminant analysis (PLS-DA) for metabolomic samples [26]. Using this approach, our metabolomic data showed classification accuracy when including only female subjects in the analysis, with 73.9±7.5% of all participants genotype (RR vs XX) predicted based on their metabolite profile. This accuracy was reached when using a set of 7 lipid metabolites for classification (Fig 2). While 4 metabolites showed reduced levels (Fig 2, Cluster A) in RR vs XX genotype females, 3 showed elevated levels (Fig 2, Cluster B), namely lyso-PC acyl C18:2, PC diacyl C38:1, and PC diacyl C40:2. Collectively, these lipid metabolites can act as a fuel source or as a cell membrane stabilizing agents. In serum, PC and lyso-PC are an important component of lipoproteins such as high-density lipoprotein (HDL) [31]. The fatty acid composition of PC is mainly determined post-synthesis by hydrolysis and re-acetylation by phospholipases and acyltransferases [32]. As these enzymes are highly active in adipose tissue, the higher % body fat observed in women might amplify the effects. The female-biased metabolomics classification is consistent with the stronger *ACTN3* genotype effects observed for both VO_2_peak and body composition.

### Pearson Correlations and Cluster Analysis of Cardiometabolic Traits

**Fig 3:**
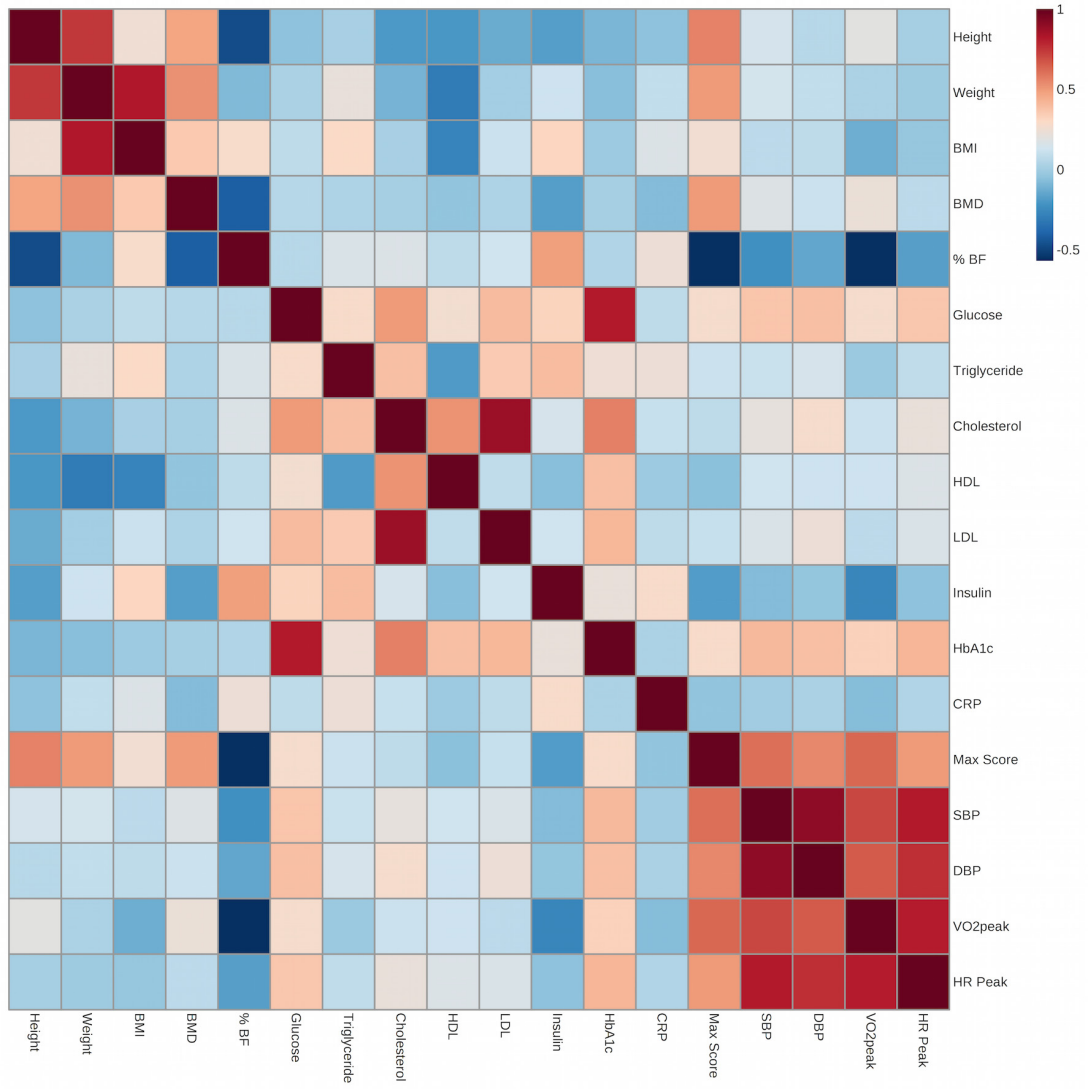
Pearson correlation matrix of 18 cardiometabolic and anthropometric variables in the University of Calgary AIMMY cohort. Positive r values are in red and negative values are in blue.

In examining multiple Pearson correlations between the cardiometabolic fitness traits in our AIMMY cohort there is a strong relationship between VO_2_peak, DBP, SBP and Peak HR, which cluster together as a block along with grip strength max score (Fig 3). This supports their statistical grouping by *ACTN3* genotype (Table 2) and identifies an association between muscle strength and cardiovascular fitness. Further analysis reveals the expected clustering of body mass traits (Weight, BMI, height, BMD) and their negative correlation with HDL-Cholesterol; The negative association of cardiovascular fitness (VO_2_peak) with cardiometabolic (%BF, Insulin, Triglycerides and CRP) and lipoprotein (HDL, LDL and Total Cholesterol) panels and the close relationship between glucose homeostasis/insulin resistance traits (Glucose, Insulin and HbA1c).

## Discussion

Whereas the *ACTN3* R577X polymorphism has been associated with muscle performance phenotypes in elite and amateur power athletes, studies linking it to cardiometabolic fitness are challenged by a lack of mechanistic evidence [5]. Herein we identified significant associations between the *ACTN3* ancestral R allele and cardiometabolic fitness in a population of healthy young adults. Specifically we have shown that *ACTN3* RR577 individuals exhibited up to 15% higher peak oxygen consumption, VO_2_ peak, compared to those of the XX genotype. Furthermore, these differences in VO_2_ peak were not associated with self-reported physical activity levels among the 3 ACTN3 genotypes (S1 Table) [19]. These findings were the opposite of what we expected given the preponderance of published research linking the RR and RX genotypes to skeletal muscle power and the XX genotype to endurance sports [2, 6]. However, in reviewing the literature it became clear that alpha-actinin 3 deficiency is not consistently advantageous in endurance athlete cohorts. Indeed, Gomez-Gallego *et al*. [33] have shown that RR/RX professional road cyclists exhibited significantly higher peak power output and ventilatory threshold than their XX counterparts. Furthermore, the R577 allele is not only overrepresented in Russian rowers but also advantageous to competition results within this same cohort [34].

Previous attempts to identify associations between *ACTN3* genotype and cardiovascular fitness tended to be ambiguous in their description of their treadmill protocol, or utilized increases in running speed to change workload [5]. As such, one interpretation of our results is that the genotype effects on VO_2_ peak are unmasked by our use of the Bruce treadmill protocol, a widely used clinical test for maximal oxygen consumption that utilizes a rapidly graded incline to increase the workload [35]. It is possible that this high treadmill incline may be sufficient to differentiate between RR and RX individuals possessing a power type muscular phenotype as compared to the α-actinin-3 deficient XX individuals. This is supported by evidence from our Pearson correlation analysis (Fig 3), which identifies an association between muscle strength and cardiovascular fitness in our AIMMY cohort. Consequently, the persistence of the R allele in elite endurance athletes likely reflects the physiological demands of contemporary endurance events wherein forceful muscle contractions are increasingly essential (i.e sprint starts and finishes) [34]. To the best of our knowledge, we are the only group to use the Bruce protocol to test for maximum oxygen consumption in relation to *ACTN3* genotype. An alternative interpretation of our VO_2_ peak results is that we may be detecting differences in submaximal oxygen consumption in RR/RX vs XX individuals due to intrinsic skeletal muscle fiber type differences. Some studies have reported RR and RX individuals as having higher proportions of type II muscle fibers compared to XX individuals [36]. Skeletal muscle with a high proportion of fast twitch fibers consumes more oxygen than slow twitch dominant muscle at submaximal stimulation despite greater overall oxidative capacity [37]. Given that the Bruce protocol is conducted in the submaximal range it is plausible that R genotype-driven differences in fiber type could be responsible for differences in submaximal exercise oxygen consumption [35].

Notwithstanding these alterative interpretations of our results, individuals with the XX genotype also displayed a significantly lower resting systolic and diastolic blood pressure than RR individuals. Given the close relationship between arterial perfusion and VO_2_ peak (Fig 3), we conducted immunoblot analysis and identified the expression of a ~100 kDa immunoreactive band corresponding to alpha-actinin 3 in human pulmonary artery smooth muscle cells (Fig 1). When it was originally identified, the expression of alpha-actinin 3 was presumed to be restricted to skeletal muscle and to a lesser extent, the brain, however recent published evidence [15] suggests a more widespread expression pattern. In addition, a cursory search of the draft (http://www.humanproteomemap.org/) identified peptide sequences unique to alpha-actinin 3 in a variety of fetal and adult human tissues and organs [38]. While our findings need to be validated *in situ* it is conceivable that changes vascular myogenic tone in response to alpha-actinin-3 deficiency could explain differences in resting blood pressure as well as altered vascular recruitment in response to an acute exercise stress test [39, 40]. Furthermore, it has not escaped our attention that the expression (or lack thereof) of alpha-actinin 3 in pulmonary artery could explain the gene-stratified differences in survival of patients with chronic heart failure [14]. Regardless of the underlying mechanism, these novel associations have clinical relevance given the wide spread use of the Bruce protocol to assess cardiac function and aerobic capacity [35].

Previous studies speculated that differences in androgen hormones could explain the sexual dimorphic effects of the *ACTN3* R577X polymorphism on muscle strength characteristics [28, 41]. As with VO_2_ peak, we identified female-driven associations between *ACTN3* genotype and body fat where females with XX genotype exhibited significantly higher % BF than individuals with the RX or RR genotypes. In fact, according to American College of Sports Medicine (ACSM) guidelines, the %BF values for the RR and RX groups were classified as “low” while the %BF for the XX group were classified as “moderate”[19]. Indeed, within our AIMMY cohort a higher percent body fat negatively correlates with VO_2_ peak and grip strength and positively with fasting insulin levels (Fig 3). As such, we believe that the interaction of *ACTN3* genotype with cardiometabolic parameters has added clinical significance.

Initially, the absence of significant genotype associations with a clinical biochemical panel (i.e Cholesterol, Triglyceride, Glucose) suggested that the metabolic differences described of *ACTN3* deficient mice may not be present in XX humans [7]. However, previous expertise with targeted metabolomics [26, 42] in our laboratory allowed us to identify a panel of 7 lipid metabolites, all phosphatidylcholines, that accurately predicted RR vs XX genotype in female subjects. Phosphatidylcholines are important components of biological membranes that have been previously shown to vary in the serum of obese adults and adolescents [43–45]. Furthermore, increased levels of lyso-PC in RR individuals may indicate elevated breakdown of phosphatidylcholines. Lyso-PC has been shown to mediate numerous physiological and cellular processes such as inflammation [46] and G-protein cell signaling [47]. It is plausible that alterations in the ratios of phosphatidylcholines may be associated with higher % body fat in female subjects or more intriguingly, the latitudinal gradient of the *ACTN3* 577X allele by contributing to metabolic efficiency and/or cellular membrane stability in colder climates [8]. While the relationship between metabolome and *ACTN3* genotype needs to be explored in a larger cohort including clinical populations (i.e heart failure, COPD) ours is the first to report metabolite differences in humans related the *ACTN3* genotype.

In summary, we have identified novel associations between the *ACTN3* R577X polymorphism peak VO_2_ and blood pressure in a population of healthy young adults. We also identified a plausible mechanism by which alpha-actinin 3 deficiency in pulmonary artery smooth muscle may influence both intrinsic aerobic capacity and the pathophysiology of heart failure. Finally, we used serum metabolomics to identify for the first time, a role for the *ACTN3* R577X polymorphism in human lipid metabolism. Taken together, these associations suggest that prospective and retrospective ACTN3 genotyping [48] may provide novel insights into human athletic performance and cardiovascular disease risk stratification.

## Supporting Information

S1 Table Comparison of Paffenbarger Survey Scores between ACTN3 genotypes. Self-reported yearly physical activity scores between ACTN3 genotypes. All analyses were performed by ANCOVA as described previously. No statistically significant differences were identified between ACTN3 genotypes RR, RX or XX (p<0.05).
(DOCX)
