# Supplementary material for: The ACTN3 R577X Polymorphism Is Associated with Cardiometabolic Fitness in Healthy Young Adults
Source: PLoS One. 2015 Jun 24;10(6):e0130644. doi: 10.1371/journal.pone.0130644 (PMC4480966; doi:10.1371/journal.pone.0130644)
Supplement: S1 Table — Self-reported yearly physical activity scores between ACTN3 genotypes. All analyses were performed by ANCOVA as described previously. No statistically significant differences were identified between ACTN3 genotypes RR, RX or XX (p<0.05). (DOCX) [file pone.0130644.s001.docx]

| Paffenbarger Survey Score | RR Genotype  n=60 | RX Genotype  n= 103 | XX Genotype  n=37 | Combined  n=200 |
| --- | --- | --- | --- | --- |
| Total Walking (miles/week) | 9.7 ± 1.3 | 8.8 ± 2.78 | 12.3 ± 2.71 | 9.7 ± 1.22 |
| Total Walking (kcal/week) | 930.90 ± 59.95 | 840.00 ± 67.32 | 1177.60 ± 192.36 | 932.11 ± 121.12 |
| Total Stair Climbing (kcal/week) | 365.10 ± 56.32 | 328.10 ± 78.25 | 331.13 ± 36.36 | 339.90 ± 45.63 |
| Light Activity (kcal/week) | 284.67 ± 42.64 | 504.06 ± 123.80 | 332.61 ± 124.12 | 406.91 ± 69.34 |
| Moderate Activity (kcal/week) | 3942.50 ± 622.83 | 3572.09 ± 400.23 | 2628.83 ± 409.77 | 3502.54 ± 287.49 |
| Vigorous Activity (kcal/week) | 4705.60 ± 1051.16 | 3304.38 ± 334.11 | 2211.48 ± 292.15 | 3501.29 ± 362.42 |
| Total Physical Activity (kcal/week) | 8932.80 ± 1444.52 | 7426.56 ± 653.46 | 5172.92 ± 618.06 | 7443.50 ± 560.39 |
| Sitting Time (hours/week) | 45.52 ± 2.45 | 44.40 ± 1.76 | 45.67 ± 2.91 | 44.97 ± 1.28 |
| Light Activity (hours/week) | 32.27 ± 1.91 | 36.32 ± 1.65 | 34.19 ± 2.66 | 34.72 ± 1.14 |
| Moderate Activity (hours/week) | 20.61 ± 1.55 | 19.77± 1.20 | 19.64 ± 1.86 | 19.99 ± 0.84 |
| Vigorous Activity (hours/week) | 12.16 ± 1.16 | 10.51 ± 0.73 | 12.22 ± 1.19 | 11.32 ± 0.56 |
| Total MET (hours/week) | 302.89 ± 6.90 | 297.60 ± 4.38 | 301.74 ± 8.32 | 299.98 ± 3.41 |

**Supplemental Table 1.** Comparison of Paffenbarger Survey Scores between ACTN3 genotypes.

**Legend:** Self-reported yearly physical activity scores between ACTN3 genotypes. All analyses were performed by ANCOVA as described previously. No statistically significant differences were identified between ACTN3 genotypes RR, RX or XX (p<0.05).
